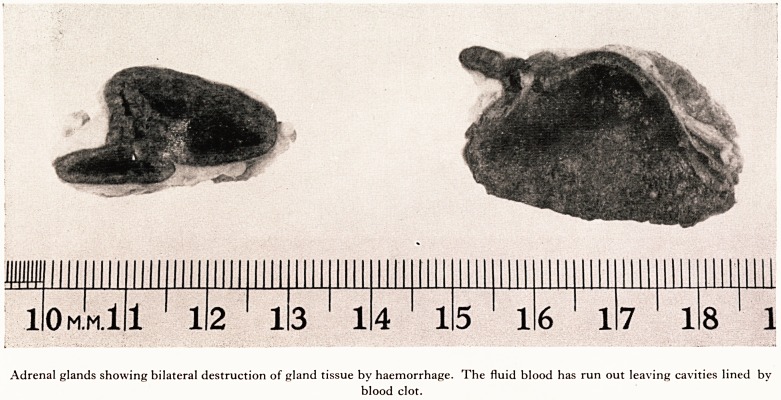# Memingococcal Septicaemia with Bilateral Adrenal Haemorrhage (Waterhouse-Friderichsen Syndrome)

**Published:** 1958-07

**Authors:** T. F. Hewer


### MEMINGOCOCCAL SEPTICAEMIA WITH BILATERAL ADRENAL HAEMORRHAGE (WATERHOUSE-FRIDERICHSEN SYNDROME)

*A Clinical Pathological Conference held at Canynge Hall on January 21st, 1958*

CHAIRMAN: PROFESSOR T. F. HEWER

*Dr. W. O. Spence:* This was a man aged 62 years whose past medical history, although not particularly relevant, was rather unusual. For many years he had complained of headaches and of attacks of pain and exquisite tenderness along the left costal margin which were only relieved by lying on the floor. He had been seen by many doctors and was regarded either as a neurasthenic or possibly as suffering from after-effects of cerebral malaria contracted during the 1914–18 war.

On October 21st, 1957 he was well although not at work—he had not, in fact, worked for ten years. The following day I was sent for and when I saw him at about 11.0 a.m., he was complaining of tremendous nausea. There was no actual vomiting but he obviously felt very much under the weather. It appeared that earlier in the day he had passed a very large motion; I did not actually see it, but he said it was a normal-coloured stool. The nausea was not relieved by passing this motion; it persisted all the time. He could hardly stand any light; this was not a true photophobia but rather that light gave him more nausea. There was little to find in the way of physical signs: his pulse was good, his colour was absolutely normal, he did not respond very well to questioning but he had always been slow in his responses. Asian influenza was epidemic at that time. I had just come from visiting several patients with it and I thought that was probably the diagnosis in this case. His temperature was not, however, raised.

Just after lunch I was called again. I found him very confused and he would not answer questions properly. His wife said that a short time before she had heard a thud and gone up to him, to find that he had passed a motion on the floor and that he seemed to think it was the right place for it. He had also vomited about a tablespoonful of blood. He now had a thready pulse and showed a typical picture of shock. His colour was about normal, by which I mean it was blanched for him: he usually had an extremely high colour but now he looked like anyone else. Abdominal palpation revealed nothing abnormal. He had always complained of tenderness in the left hypochondrium but this was not present at 2.30 that afternoon. To combat the shock I gave him morphia gr. $\frac{1}{4}$ and went back at four o'clock when I found he was asleep. I saw him but did not awaken him. His general condition had improved. His pulse was of much better volume and not so rapid.

Later, I was summoned to see another urgent case (from one of these conferences!) after which I called to see him again at 7 p.m. The change in his condition was remarkable. His colour was dusky although he was not truly cyanosed. His pulse was almost imperceptible. He was cold and sweating. In view of the fact that he had had a small haematemesis I thought he must be bleeding internally so I called in Mr. Bartlett who saw him at 7.45 p.m. but decided, after going over the case carefully, that it was not a surgical problem; we decided together to call Dr. Mather. Between 7.45 p.m. and 8.10 p.m. the patient developed generalised purpura with true cyanosis seen in the fingers, nose and toes.

*Dr. H. G. Mather:* When I arrived at about 8.10 p.m. it was quite extraordinary how ill this man was and how quickly he had deteriorated. There was generalised purpura of the skin and conjunctivae. He was a desperately ill man. His systolic blood pressure was not above 40 mm.Hg. and his pulse rate was 120 per minute. There was just a suggestion of neck stiffness. His plantar reflexes were flexor and there were no other physical signs. I thought the most likely diagnosis was an overwhelming septicaemia with adrenal haemorrhage causing the shock, low blood pressure and purpura. I took blood for a blood culture and gave him an intravenous injection of sulphadimidine (2 Gms), tetracycline (500 mgm.) and hydrocortisone (100 mgm.) and intramuscular penicillin (1 mega unit). The main line of treatment seemed to me to be to replace the adrenal hormones and to knock out whatever organism was causing the septicaemia; this seemed most likely to be the meningococcus. Examination of the blood revealed a white blood count of 8,700 per cu.mm. This is not high but the important feature was that the differential count showed large numbers of primitive polymorphs (metamyelocytes 26 per cent, stabs 18 per cent, polymorphs 46 per cent, lymphocytes 8 per cent, monocytes 2 per cent). This strongly suggested a very severe infection.

An hour later his systolic blood pressure was 70 mm.Hg. Another intravenous injection of sulphadimidine and hydrocortisone was given together with "mephine" to try and raise the blood pressure. By then we had done as much as we could to get this man over his illness. We felt he was much too ill to risk moving him to hospital; he would almost certainly have died in the ambulance. If he survived another twelve hours we might get him into hospital the next day.

However, he died at 1.45 a.m. the next morning, at home. To my mind it is this that makes him such an interesting case. As a rule the cases we discuss here have been thoroughly investigated in hospital but here you can see the difficulties experienced by doctors who have to treat patients in their own homes.

*Question:* Was this man shivering at the time you saw him?

*Dr. Mather:* No, I did not see any sign of this.

*Mr. R. V. Cooke:* I see from the history that he was seen by many consultants, many of whom had regarded him as neurotic. Did any of the earlier symptoms have any relation to the cause of death? Could a diagnosis of a fatal disease have been made earlier?

*Dr. Mather:* We have not yet, of course, heard the postmortem findings but if you accept our clinical diagnosis of an acute septicaemia with suprarenal haemorrhage then it would appear to be something new and quite unrelated to whatever chronic disease he had suffered from for so many years.

*Question:* Was the purpura an early sign in this case?

*Dr. Spence:* No, it came on during the evening, but it advanced very rapidly so that before he died his hands, nose and feet were almost black.

*Dr. N. J. Brown:* (presenting the autopsy findings). This man, as you have heard, died at home but Dr. Spence asked me to arrange for a postmortem as we sometimes do in these cases and so he came to see me at Southmead Hospital as a kind of out-patient! He was a well-nourished, heavily built man with extensive purpura over his face, neck, trunk and limbs. He was deeply cyanosed and had been bleeding from the mouth. We were soon able to confirm the clinical diagnosis of adrenal haemorrhage but first of all I will give you the other findings.

There were a few old adhesions at the base of the left lung; otherwise the serous cavities were normal. The heart was soft and flabby with dilatation of all its chambers, which was consistent with septicaemia. The lungs showed oedema and congestion. The spleen was slightly soft and pale but it was not the typical picture of a "septic spleen". There were no enlarged lymph nodes. The liver was congested and showed mottling of its cut surface; there was also a curious atrophy of the left lobe. The stomach contained a little blood and there was a chronic ulcer in the first part of the duodenum which appeared to have been bleeding a little. There was some blood in the upper part of the small intestine. Bearing in mind the strange attacks of pain which Dr. Spence has told you about, I tried to find some cause for them. It was possible that it might have been referred pain from this duodenal ulcer although one would not have expected pain from that origin to be under the left ribs. Another possible explanation was to be found in the fact that his pelvic colon was exceptionally large and was greatly distended by gas. It extended, in fact, right up to the region of the spleen and was kinked at its lower end although there was no apparent organic obstruction. It could be that his pains had been due to "wind" due to this abnormal colon. I could find no better explanation.

The kidneys showed a little blurring of their pattern and were slightly swollen. The thyroid and pituitary were normal. The brain appeared slightly swollen with some hyperaemia of the meningeal vessels but there was no real macroscopic evidence of meningitis. The adrenals were greatly enlarged (see Plate III) and the centre of each was completely destroyed and filled with fluid blood leaving only a thin rim of cortical tissue around the edge. This man died therefore, as was clinically diagnosed, from massive bilateral adrenal haemorrhage.

This condition is known as the Waterhouse-Friderichsen syndrome. It was first described by neither of these two authors but by Little in 1901. Waterhouse gave the first detailed account in 1911, at which time he was pathologist to the Royal United Hospital at Bath. He thought it was due to smallpox. Friderichsen described it in 1918. Since then many cases have been recorded, most of which have been associated with septicaemia, of which meningococcal septicaemia is by far the most common.

In this case I took swabs from various organs and gave them to Dr. Crowther. From the lung, adrenal and brain he succeeded in growing gram-negative diplococci which were proved by fermentation reactions to be meningococci. When you consider that this man had been heavily treated with drugs to which these organisms are sensitive and that moreover he had been dead for thirty-six hours before I carried out a postmortem examination, you will realise how numerous these organisms must have been during life for them to have been still recoverable at autopsy.

Histologically the adrenals showed gross destruction of the medulla and inner part of the cortex by haemorrhage. The surviving cortical tissue showed intense capillary congestion and small petechial haemorrhages. The brain showed no microscopical evidence of meningitis.

In conclusion I must tell you about a rather surprising finding. In the lung there were a few pale subpleural nodules. Histologically these were composed of follicular collections of epithelioid cells and giant cells; the lesions were sharply circumscribed, there was no caseation and no acid-fast bacilli were found. These are in fact the lesions of Boeck's sarcoidosis and when the liver was examined histologically the tiny left lobe was found to be absolutely full of similar lesions. Whether this had anything to do with the atrophy of the left lobe of the liver I do not know. What is certain is that this man suffered from sarcoidosis in addition to all his other troubles. It is difficult however to see any connection between the chronic disease—sarcoidosis—and the fulminating meningococcal septicaemia which killed him.

*Dr. F. J. W. Lewis:* I think there may well be such a connection. Sarcoidosis involves the reticulo-endothelial system and, although I am not saying that it is so, it could be that, in this case, the septicaemia became overwhelming because the very system which provides the mechanism for combating infection had been partially knocked out by sarcoidosis.

*Dr. O. C. Lloyd:* Is it a fact that people with sarcoidosis are more liable to get septicaemia than normal people?

*Dr. Lewis:* I don't know, but experimentally if you knock out the reticulo-endothelial system you do get this effect.

*Prof. T. F. Hewer:* Was the rest of the reticulo-endothelial system affected by sarcoid? What about the spleen and lymph nodes?

*Dr. Brown:* No. There were no enlarged lymph nodes and a section of the spleen showed no sarcoid.

*Prof. R. Milnes Walker:* Dr. Brown pointed out that there were a lot of sarcoid lesions in the liver and that this might have accounted for what he called atrophy of the left lobe. Are we sure that this is really a true atrophy? Do we know that the left lobe was ever any bigger?

*Dr. Brown:* We cannot be sure that it was truly atrophy and perhaps I used the

PLATE III

Adrenal glands showing bilateral destruction of gland tissue by haemorrhage. The fluid blood has run out leaving cavities lined by blood clot.

term without real justification. It is only fair to say that I have seen a number of cases at autopsy like this with a small left lobe to the liver and they have not had sarcoidosis or anything else to account for it.

*Mr. R. V. Cooke:* Occasionally at operation we find that part of the liver is virtually missing in a perfectly healthy patient. It is just a normal variation.

*Dr. Brown:* I only said the sarcoidosis *might* have had something to do with it. I have no strong feelings in the matter. Shall we agree to say that the left lobe of the liver was smaller than usual?

*Dr. Mather:* Dr. Crowther also managed to grow meningococci from the blood which I took for culture although this was not put straight into broth. This indicates an extremely heavy infection.

*Dr. Lloyd:* Were they sensitive to the antibiotics already given?

*Dr. J. E. Cates:* Sulphadimidine is excellent treatment for most meningococcal infections but it only inhibits the organisms; it is not bactericidal. Penicillin is bactericidal but it might not have been able to reach the organisms for the circulation was failing and this drug was not given intravenously.

*Dr. Spence:* Ought one to have been able to make the diagnosis earlier in this case?

*Dr. Mather:* I do not think so. The course was very rapid making early diagnosis extremely difficult.

*Prof. A. V. Neale:* Would you ever expect anyone to get better once this massive adrenal haemorrhage had occurred?

*Dr. Mather:* Cases have been reported which have recovered on the sort of intensive therapy which we gave this man. Some had adrenal insufficiency afterwards and had to be maintained on adrenal hormones.

*Question:* Is there any known source of infection in this case?

*Dr. Mather:* Meningococci are found in the throat and nasopharynx in a certain number of normal carriers. For some unknown reason the barrier sometimes breaks down and the infection spreads in the blood stream.

*Dr. Lewis:* The explanation usually given for the purpura in these cases is that there occurs a type of Schwartzman reaction mentioned by Professor Neale at a previous meeting. In this there are localised areas of sensitization and the toxic circulating bacteria act as a "shocking" agent producing necrosis and haemorrhage. The time lag between the sensitization and the "provoking" dose can be very short—twenty-four hours or less.

## Figures and Tables

**Figure f1:**